# *VSX1* gene analysis in keratoconus

**Published:** 2010-11-16

**Authors:** Mukesh Tanwar, Manoj Kumar, Bhagabat Nayak, Dhananjay Pathak, Namrata Sharma, Jeewan S. Titiyal, Rima Dada

**Affiliations:** 1 Laboratory For Molecular Reproduction and Genetics, Department of Anatomy, All India Institute of Medical Sciences, Ansari Nagar, New Delhi, India; 2 Dr. R.P. Centre for Ophthalmic Sciences, All India Institute of Medical Sciences, Ansari Nagar, New Delhi, India

## Abstract

**Purpose:**

To screen the visual system homeobox 1 (*VSX1*) gene in keratoconus patients.

**Methods:**

The enntire coding region of *VSX1*, including intron-exon boundaries were amplified in keratoconus cases (n=50) and controls (n=50). All sequences were analyzed against the ensemble sequence (ENSG00000100987) for *VXS1*.

**Results:**

Sequencing analysis showed four alterations (p.A182A, p.R217H, p.P237P, and g.25059612C>T) in *VSX1* of which g.25059612C>T (in intron 2) was found to be novel. Of these four, p.A182A and p.P237P were present in both cases as well as controls while p.R217H and g.25059612C>T were limited to cases only. All these changes were non-pathogenic.

**Conclusions:**

In our study no pathogenic *VSX1* mutation was identified. The role of *VSX1* in the pathogenesis of keratoconus is still controversial. *VSX1* mutations are responsible for a very small fraction of all observed keratoconus cases. The absence of pathogenic mutations in *VSX1* in our patients indicates that other genetic loci like 13q32 as suggested by a recent study may be involved in the pathogenesis of this disorder.

## Introduction

Keratoconus (KTCN; OMIM 148300) is a bilateral, non-inflammatory, and progredient corneal ectasia [1]. There is no specific treatment for keratoconus except corneal transplantation. It has an estimated incidence between 1/500 to 1/2,000 persons throughout the world. The disease usually arises in the teens and stabilizes in the third and fourth decade of life [2]. It occurs with no ethnic or gender preponderance and causes significant visual impairment. Most cases of keratoconus are sporadic but some (5%–10%) have a positive family history [2,3]. In such cases both autosomal dominant and recessive patterns of inheritance have been documented [4-6]. The exact pathogenesis of keratoconus is still unknown. Genome-wide linkage analyses has identified several chromosomal loci and genes that may be associated with keratoconus [6-9]; however, some were eventually excluded [10,11] while for others a conclusive association with the disease is yet to be established. Mutations in the visual system homeobox 1 (*VSX1)* gene in keratoconus have been reported in different studies [12-15].

*VSX1* is a member of the Vsx1 group of vertebrate paired-like homeodomain transcription factors. It has been localized to human chromosome 20p11-q11. Initially *VSX1* was chosen for screening mutations in posterior polymorphous corneal dystrophy (PPCD) and keratoconus [8]. *VSX1* is considered important in ocular development and is particularly involved in the developing cornea. Expression in human was demonstrated in embryonic craniofacial, adult corneal, and adult retinal cDNA libraries [16]. *VSX1* mRNA has been found in the outer tier of the inner nuclear layer of the human retina and the cornea [17]. Mutations in this gene are also associated with posterior polymorphous dystrophy. *VSX1* is highly conserved across many species [18-20]. Several mutations, such as p.D144E, p.G160D, p.P247R, p.L159M, p.R166W, and p.H244R have been reported by various groups [14,17,21] but a definite pathogenic role of these mutations in keratoconus is not yet established. In this study we present the results of *VSX1* gene analysis in 50 keratoconus patients and controls from north India.

## Methods

### Patient selection and DNA isolation

The research followed the tenets of the Declaration of Helsinki in the treatment of the subject reported herein. The study was approved by institutional review board (IRB # IRB00006862) of the All India Institute of Medical Sciences (AIIMS) and all participants gave their written informed consent. A total of fifty keratoconus patients (Table 1) presented (during April 2009 to April 2010) at the Dr. R. P. Centre for Ophthalmic Sciences (AIIMS, New Delhi, India) were enrolled in this study. Clinical evaluation involved Ultrasonic Pachymetry, videokeratography (VKG), Orbscan, visual testing, fundoscopy, slitlamp-biomicroscopy, and retinoscopy. Of these patients, 29 were males and 21 were females. The mean age of presentation was 18.2 years. Diagnosis of keratoconus involved the presence of characteristic topographic features, such as inferior or central corneal steepening, or an asymmetric bowtie pattern with skewing of the radial axes, and the presence of one or more of the following characteristic clinical features in one or both eyes: conical corneal deformation, munsen sign, corneal stromal thinning, a Fleischer ring or Vogt striae. All cases were sporadic without any family history.

**Table 1:** Clinical phenotype of keratoconus patients.

** **	** **	** **	**Visual Acuity in Snellen’s chart**		**Munsen sign**		**Vogt's striae**		**Hydrops**		**Scarring**		**Keratometry in VKG (in Diopters)**		**Ultrasonic Pachymetry (in μm)**	
**Patient ID**	**Age in years**	**Sex**	**OD**	**OS**	**OD**	**OS**	**OD**	**OS**	**OD**	**OS**	**OD**	**OS**	**OD**	**OS**	**OD**	**OS**
KC1	20	F	6/12	6/12	₊	+	-	-	-	-	-	-	45.62	46.37	490	414
KC2	12	M	6/60	6/60	+	+	₊	+	+	+	+	+	56	52	344	347
KC3	22	F	6/12	6/6	+	+	₊	+	+	-	+	-	56.6	49.5	396	412
KC4	20	M	6/12	6/9	+	+	+	+	+	-	+	-	48.5	45	406	498
KC5	20	M	6/24	6/6	+	-	+	-	+	-	+	-	54	46.5	410	520
KC6	19	M	6/12	6/12	+	+	+	+	-	-	-	-	46.5	46.5	501	488
KC7	18	M	618	6/60	+	+	+	+	-	+	-	+	52.12	Distorted	480	344
KC8	14	F	6/36	6/24	+	+	+	+	+	+	+	₊	>52	52	336	346
KC9	20	M	6/9	6/9	+	+	+	+	-	-	-	₋	50.5	49.1	460	436
KC10	22	M	6/24	6/9	+	-	+	-	-	-	-	-	52	47.1	344	420
KC11	19	M	6/18	6/18	+	+	+	+	+	+	+	-	54.75	56	410	400
KC12	17	M	6/12	6/18	+	+	+	+	-	-	-	-	51.12	49.87	419	414
KC13	20	F	6/9	6/12	+	+	+	+	-	-	-	-	48.5	51.25	440	456
KC14	10	M	6/12	6/9	+	+	+	-	+	-	+	-	61.12	48.75	402	512
KC15	22	F	6/12	6/12	+	+	-	+	-	-	+	+	48.5	48	502	486
KC16	18	F	6/6	6/60	-	-	-	+	-	+	-	+	Distorted	49.5	510	265
KC17	22	M	6/6	6/9	+	+	+	+	-	-	-	-	49.5	51.75	399	353
KC18	15	M	6/9	6/18	+	+	+	+	+	-	-	-	48.25	48.75	485	493
KC19	20	F	6/60	6/60	+	+	+	+	+	+	+	+	Distorted	Distorted	230	330
KC20	20	F	2/60	6/60	+	+	+	-	+	-	+	-	Distorted	41.37	341	510
KC21	16	M	6/12	6/9	+	+	+	+	-	-	-	-	48	48.25	484	496
KC22	18	M	3/60	6/12	+	+	+	+	+	-	+	-	Distorted	54.5	330	372
KC23	20	F	6/12	6/12	+	+	+	+	-	-	-	-	49	49	420	456
KC24	21	M	6/9	69	+	+	+	-	-	-	-	-	46.5	61.25	510	445
KC25	18	F	6/36	6/24	+	+	+	+	+	+	+	+	54.87	55.25	294	297
KC26	8	M	6/18	6/18	+	+	+	+	+	+	+	+	49.25	68	348	330
KC27	15	M	6/12	6/12	+	+	-	+	-	-	-	-	48.25	53.25	456	400
KC28	15	M	6/12	6/24	+	+	+	+	-	-	-	-	53	58.5	430	398
KC29	17	F	6/9	6/12	+	+	+	+	-	+	-	+	49.5	57	446	398
KC30	11	M	6/18	6/12	+	+	+	+	+	-	+	-	62.25	48.25	314	423
KC31	19	F	6/18	6/6	+	+	+	-	-	-	-	-	50.37	46.25	419	338
KC32	20	F	6/9	6/12	+	+	-	+	-	-	-	-	45	48.75	520	510
KC33	22	M	6/9	6/9	+	+	-	+	-	-	-	-	47	48.75	530	490
KC34	16	F	6/9	1/60	+	+	+	+	+	+	+	+	45.5	67.28	480	290
KC35	20	F	6/12	6/12	+	+	+	+	-	-	-	-	48.12	48.5	498	480
KC36	17	F	6/60	6/12	+	+	+	-	-	-	-	-	56.5	48	356	423
KC37	10	M	6/12	6/12	+	+	+	-	-	-	-	-	52.9	46.5	433	460
KC38	25	M	6/9	6/12	+	+	+	+	+	-	+	-	68	51	310	424
KC39	21	F	6/6	6/24	-	+	-	+	-	-	-	-	46.12	58.75	474	378
KC40	18	M	6/9	6/9	+	+	+	+	-	-	-	-	48.5	48.15	456	478
KC41	26	F	6/12	6/12	+	+	+	+	-	-	-	-	57.5	52.62	467	490
KC42	29	M	6/12	6/18	+	+	+	+	-	-	-	-	53.5	51	486	496
KC43	22	M	6/9	6/9	+	+	+	-	-	-	+	-	54	45.37	320	400
KC44	14	M	6/60	6/6	+	-	+	-	+	-	+	-	58.37	42.5	386	524
KC45	23	M	6/6	6/24	+	+	+	+	-	-	+	-	54	49.75	326	328
KC46	16	F	6/18	6/24	+	+	+	-	-	-	-	+	47.13	53	480	432
KC47	11	M	6/18	6/60	+	+	+	+	+	+	+	+	54.87	Distorted	332	Poor echo
KC48	14	F	6/36	6/6	+	-	+	-	-	-	-	-	52	41.87	345	580
KC49	18	M	6/12	6/18	+	+	+	+	-	-	-	+	48	50	456	438
KC50	20	F	6/9	6/24	+	+	+	+	-	-	-	-	49.25	56.12	341	465

Key: M=male; F=female; OD=right eye; OS=left eye; +=positive; -=negative; VKG=videokeratography

All keratoconus cases secondary to causes like trauma, surgery, Ehlers Danlos syndrome, Osteogenesis Imperfecta, and pellucid marginal degeneration were excluded from the study.

After informed consent, detailed personal, medical and occupational history was collected and a family tree up to three generations was drawn. Fifty ethnically matched normal individuals without any ocular disorder were enrolled as controls. Health information was obtained from controls through the questionnaire; all underwent ophthalmological examination and blood sample (5 ml) was collected in EDTA (EDTA) vacutainers (Greiner Bio-One GmbH, Frickenhausen, Germany) from patients and controls for DNA extraction. DNA was extracted from whole blood samples of all patients and controls using the phenol-chloroform method.

### PCR and DNA sequencing

All the coding regions of *VSX1* including exon-intron junctions were amplified using a set of five oligonucleotide primers (Table 2). Each reaction was performed in a 25 µl mixture containing 0.2 µM each primer, 0.5 U Taq DNA polymerase (Biogene, New Delhi, India), 2.5 µl 10× PCR buffer (Biogene) with 2.5 mM MgCl_2_, and approximately 100 ng genomic DNA. Thermal cycling was performed in a thermal cycler (My Cycler; Biorad, Gurgaon, India) under the following conditions: initial denaturation for 3 min at 95 °C; 35 cycles of 94 °C for 30 s, 55 ^0^-60 °C for 45 s, 72 °C for 60 s; and a final extension for 10 min at 72 °C.

**Table 2:** Primers used for *VSX1* gene amplification.

**Exon**	**Forward primer (5′-3′)**	**Reverse primer (5′-3′)**	**Product size (bp)**
1	CAGCTGATTGGAGCCCTTC	CTCAGAGCCTAGGGGACAGG	599
2	GCACTAAAAATGCTGGCTCA	GCCTCCTAGGAACTGCAGAA	393
3	CATTCAGAGGTGGGGTGTT	TCTTGTGGTGCCTTCAGCTA	419
4	GATCATGCTCGGGAGAGAAG	CGTTGCTTTGCTTTGGAAAT	394
5	CCCCAGAGATAGGCACTGAC	TGGACAATTTTTGTCTTTTGG	495

All PCR products were analyzed on 1.8% agarose gel stained with ethidium-bromide (EtBr; 10 mg/ml). Agarose gels were analyzed using gel documentation system (Applied Biosystems, Carlsbad, CA). Amplified PCR products were purified using gel/PCR DNA fragments extraction kit (DF100; Geneaid Biotech Ltd., Sijhih City Taiwan). The purified PCR products were sent for sequencing at MCLAB (Molecular Cloning Laboratories, South San Francisco, CA)

Nucleotide sequences were compared with the *VSX1* ensembl reference sequence.

### Insilico analysis of missense mutations

Two homology based programs PolyPhen (Polymorphism Phenotyping) and SIFT (Sorting Intolerant From Tolerant) analysis tool were used to predict the functional impact of missense changes identified in this study.

PolyPhen structurally analyzes an amino acid polymorphism and predicts whether that amino acid change is likely to be deleterious to protein function [22,23]. The prediction is based on the position-specific independent counts (PSIC) score derived from multiple sequence alignments of observations. PolyPhen scores of >2.0 indicate the polymorphism is probably damaging to protein function. Scores of 1.5–2.0 are possibly damaging, and scores of <1.5 are likely benign.

SIFT is a sequence homology-based tool that sorts intolerant from tolerant amino acid substitutions and predicts whether an amino acid substitution in a protein will have a phenotypic effect [24,25]. SIFT is based on the premise that protein evolution is correlated with protein function. Positions important for function should be conserved in an alignment of the protein family, whereas unimportant positions should appear diverse in an alignment. Positions with normalized probabilities less than 0.05 are predicted to be deleterious and, those greater than or equal to 0.05 are predicted to be tolerated.

We have also used improved Splice Site predictor tool [26] for prediction the effect of an intronic nucleotide change on splicing.

## Results

DNA sequencing analysis of 50 patients and 50 controls revealed a total of four nucleotide changes (Table 3) of which one was novel and 3 have been previously reported. Details of these changes are given below.

**Table 3:** *VSX1* sequence variants observed in this study.

**Nucleotide Change**	***VSX1* transcript ID**	**Protein alteration**	**Exon/ UTR/ intron**	**Patients (n=50)**	**Controls (n=50)**	**Reference/ SNP ID**	**Polyphen/SIFT prediction**
g.25059546A>G (rs12480307)	NM_014588	p.A182A	Exon 3	25/50	29/50	[20]	–
g.25059442G>A (rs6138482)	NM_0199425	p.R217H	Exon 3	1/50	Absent	[20]	Non-pathogenic
g.25059381T>A (rs56157240)	NM_0199425	p.P237P	Exon 3	18/50	14/50	[20]	–
g.25059612C>T	–	–	Intron 2	3/50	Absent	Novel	–

### Alanine182Alanine (p.A182A)

**Figure 1:**
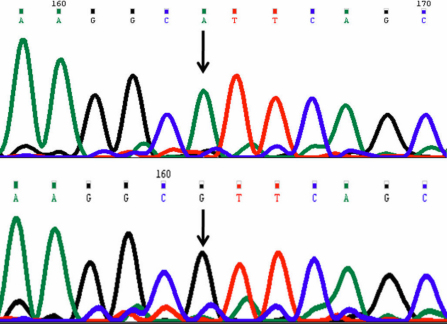
DNA sequence chromatogram of *VSX1* equivalent to codon 181 to 184. **A**: The reference sequence derived from control is shown. **B**: Sequence derived from keratoconus patient shows homozygous A>G nucleotide change which predicts a codon change GCA>GCG and synonymous change p.A182A.

In this mutation a single nucleotide adenine (A) was replaced by guanine (G) at g.25059546 (rs12480307); c.546; codon 182 resulted in codon change GCA>GCG resulting in synonymous change p.ala182ala (p.A182A; Figure 1). This change was present as homozygous change in 25 cases and 29 controls.

### Arginine217Histidine (p.R217H)

**Figure 2:**
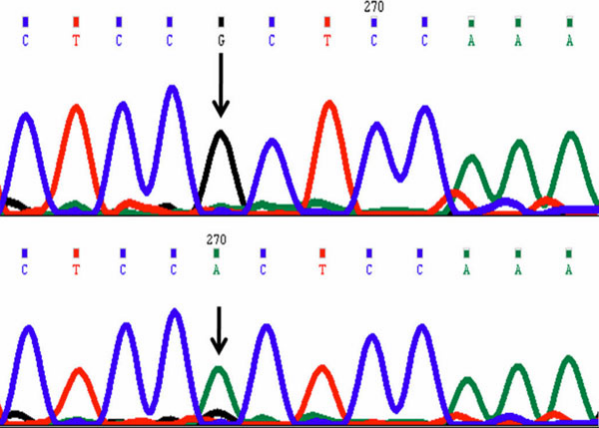
DNA sequence chromatogram of *VSX1* equivalent to codon 217 to 219. **A**: The reference sequence derived from control is shown. **B**: Sequence derived from keratoconus patient shows homozygous G>A nucleotide change which predicts a codon change CGC>CAC and non-synonymous change p.R217H.

In this mutation a single nucleotide thymine (T) was replaced by adenine at position g.25059442 (rs6138482); cDNA position c.650; codon 217. This change resulted in a codon change from CGC>CAC resulting in non-synonymous change p.arg217his (p.R217H; Figure 2) in protein. This change was present in only one case and was homozygous but was absent in controls.

### Proline237Proline (p.P237P)

**Figure 3:**
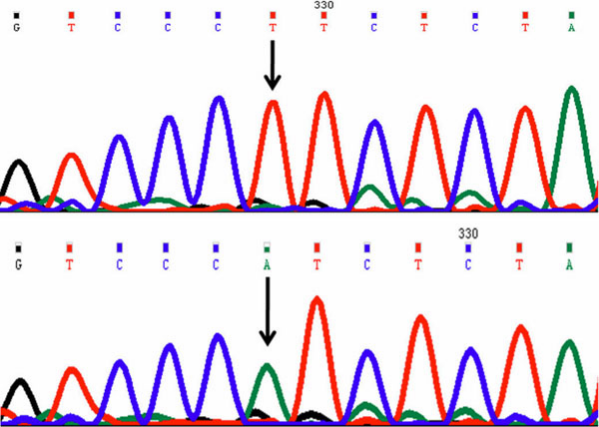
DNA sequence chromatogram of *VSX1* equivalent to codon 236 to 239. **A**: The reference sequence derived from control is shown. **B**: Sequence derived from keratoconus patient shows homozygous T>A nucleotide change which predicts a codon change CCT>CCA and synonymous change p.P237P.

In this mutation a single nucleotide T was replaced by A at position g.25059381 (rs56157240); c.711; codon 237 resulted in a codon change CCT>CCA which predicts a synonymous change p.pro237pro (p.P237P; Figure 3). This change was present in 18/50 cases (7 were homozygous and 11 were heterozygous) being also present in 14/50 controls (9 were homozygous and 5 were heterozygous).

### Cytosine to Thymine in intron 2

**Figure 4:**
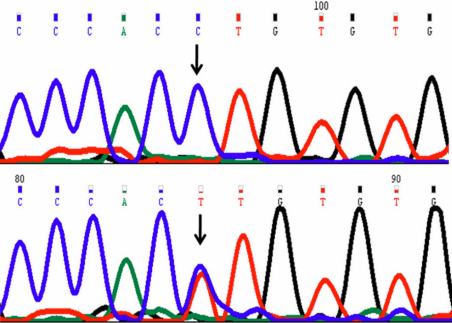
DNA sequence chromatogram of *VSX1* equivalent to g.25059617 to g.25059606. **A**: The reference sequence derived from control is shown. **B**: Sequence derived from keratoconus patient shows heterozygous C>T nucleotide change in intron 2 at g.25059612.

A novel single nucleotide change C>T at g.25059612 (Figure 4) was present in three cases but absent in controls. Alteration is located in 2nd intron (IVS3–24C>T). This change was registered at GenBank with accession number GU471016.

PolyPhen and SIFT analysis of p.R217H showed that it is non-pathogenic (SIFT score is >0.05 and PSIC score is <1.5).

Improved splice site predictor tool analysis of g.25059612C>T showed that this location (g.25059612) is not present at splice site and may not create splicing error in *VSX1* mRNA. Conclusively, no pathogenic change was identified in our patients.

## Discussion

In this study we analyzed *VSX1* in 50 unrelated keratoconus patients and controls from north India. In our patient’s, males were affected more than females. Mutations in *VSX1* gene have been identified in association with keratoconus [12,14,15,17,21]. Human *VSX1* is a member of the CVC domain containing paired-like class of homeoproteins. *VSX1* expression in humans was detected in embryonic craniofacial, adult retinal, and adult corneal tissues [17,27]. The role of *VSX1* in keratoconus is still ambiguous. Previous studies have shown that the pathogenesis of keratoconus is very complex and several genes and gene environmental interactions play a critical role in disease prognosis. In fact, *VSX1* may have a pleiotropic action among the tissues of the cornea leading to keratoconus in some cases as observed for the transforming growth factor (*TGFBI*) gene; which causes four distinct autosomal dominant corneal diseases [28].

In this study, four sequence variations were detected of which 3 have been previously reported [15,20] and one was novel. SIFT and PolyPhen analysis of p.217H showed that it to be a non-pathogenic change. Similar findings have already been reported in European population [15].

The *VSXI* variants reported in various studies include p.L17P, p.D144E, p.N151S, p.L159M, p.G160V, p.G160D, p.R166W, p.Q175H, p.H244R, and p.P247R [12-15,17,21]. Some of these were initially reported to be pathogenic but their pathogenicity could not be confirmed as some of these variants were observed in unaffected individuals also. He’on and associates [14] identified a compound heterozygous change with p.P247R and p.G160D and reported p.G160D to be pathogenic and p.P247R to be nonpathogenic. Another study reported p.P247R as a pathogenic change because it was found to be co-segregating with keratoconus. Similarly, p.D144E mutation was initially reported as pathogenic [20,21,] but subsequent studies identified its presence in unaffected individuals and suggested this to be a non-pathogenic polymorphism [29,30]. The variants p.R166W, p.H244R, and p.L159M have been identified in keratoconus patients but these changes did not segregate with the disease phenotype in their family members and hence were not considered sufficiently significant to support a pathogenic role in keratoconus [31]. Similarly, variants p.G160V and p.N151S have been identified in patients from the Korean population [12] but these changes have not been reported in other populations.

In a recent study (2009) from India, *VSX1* was screened in 66 keratoconus cases and a potentially pathogenic change (p.Q175H) was identified in one case only [13]. In this study,  the second from India, no pathogenic change  was indentified in *VSX1*. Similar results have been published recently [29,32,33]. So lack of possibly pathogenic changes in *VSX1* gene in keratoconus patients suggests that mutations of *VSX1* could only be responsible for a very small fraction of all observed cases and need to be investigated in different populations. This also suggests that other genetic loci like 13q32 as suggested by Gajecka et al. [34] may be involved in the pathogenesis of keratoconus.
